# The threshold of an excitable system serves as a control mechanism for noise filtering during chemotaxis

**DOI:** 10.1371/journal.pone.0201283

**Published:** 2018-07-30

**Authors:** Sayak Bhattacharya, Pablo A. Iglesias

**Affiliations:** 1 Department of Electrical and Computer Engineering, Whiting School of Engineering, Johns Hopkins University, Baltimore, Maryland, United States of America; 2 Department of Cell Biology, School of Medicine, Johns Hopkins University, Baltimore, Maryland, United States of America; East Carolina University Brody School of Medicine, UNITED STATES

## Abstract

Chemotaxis, the migration of cells in the direction of a chemical gradient, is of utmost importance in various biological processes. In recent years, research has demonstrated that the underlying mechanism that controls cell migration is an excitable network. One of the properties that characterizes excitability is the presence of a threshold for activation. Here, we show that excitable systems possess noise filtering capabilities that enable faster and more efficient directed migration compared to other systems that also include a threshold, such as ultrasensitive switches. We demonstrate that this filtering ability is a consequence of the varying responses of excitable systems to step and pulse stimuli. Whereas the response to step inputs is determined solely by the magnitude of the stimulus, for pulse stimuli, the response depends on both the magnitude and duration of the stimulus. We then show that these two forms of threshold behavior can be decoupled from one another, allowing finer control in chemotaxis. Finally, we use a simple model of chemotaxis to demonstrate that cells that rely on an excitable system display faster and more effective directed migration that a hypothetical cell guided by an ultra-sensitive switch.

## Introduction

The response of many biological systems is based on whether the level of a stimulus is above or below a given threshold. For example, *Xenopus oocytes* undergo apoptosis based on an all-or-none switch-like response from the MAPK cascade signaling pathway [1]. Similarly, the entry into mitosis in budding yeast is also controlled by positive feedback loops that generate switch-like decision making [2, 3]. The importance of switches to biological function was likely one of the reasons that the development of genetic switches was one of the first steps in synthetic biology [4].

The biological signaling pathway that allows amoeboid cells, such as the social amoebae *Dictyostelium discoideum* or human neutrophils, to move also appears to employ switch-like behavior. When placed in a gradient, the side of the cell facing the chemoattractant source shows high level of activity as measured either in terms of actin polymerization or by using fluorescent signaling biosensors. The rear of the cell, however, shows significantly lesser activity, suggesting that gradient information is processed through a threshold [5].

Until recently, the nature and the biochemical circuitry that implements this threshold have been elusive, though it is now believed to be a consequence of an excitable network that regulates actin polymerization leading to pseudopod formation [6–8]. Excitable systems are a class of dynamical systems that display an all-or-nothing response with distinct on and off states. Specifically, these systems exhibit large responses to suprathreshold stimuli while remaining quiescent under subthreshold stimuli. Apart from this characteristic, excitable systems also display a refractory period following a response, during which the system does not respond even to suprathreshold stimuli while it recovers excitability. There is now ample evidence that an excitable signal transduction network controls random and directed migration in many different cell lines [9]. In the absence of directional cues, stochastic perturbations in the concentration of various signaling molecules likely trigger the excitable system, which in turn leads to the creation of the actin-rich pseudopods that propel cells [6]. The presence of a chemoattractant source generates persistent, graded external cues that selectively alter the threshold spatially to direct the movement of these cells [10].

The different responses of excitable systems to step, pulse and ramp stimuli have allowed for the characterization of the threshold for activation in neural systems [11]. A similar characterization has been done on cell migration models to classify their excitable dynamics [12]. Based on such observations, Izhikevich suggested that excitable systems have a *soft* threshold described by a *manifold* [11] in phase space that has to be crossed to elicit a response. This is in contrast to a hard threshold of a typical switch, that presents in the form of a particular value which the input magnitude has to surpass in order for the system to respond.

Here, we show that the presence of a threshold manifold enables excitable systems to serve as more robust noise filters when compared to hard thresholds. Moreover, in the context of cell migration, the threshold criteria that arise from different input-types allow for greater tunability in the response of cells to spatially graded stimuli, in turn enabling efficient and controlled directed migration.

## Mathematical background

**Fig 1:**
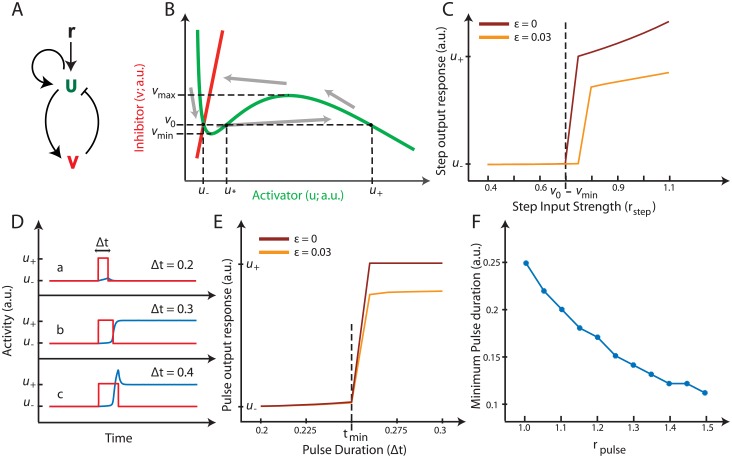
Model dynamics and thresholds. A. The two species activator (*u*)-inhibitor (*v*) system with external input *r*. B. Phase-plane representation with the activator and inhibitor nullclines in green and red respectively with the critical concentrations marked. A typical suprathreshold response trajectory is shown by the grey arrows. C. Computing the response (final value) with increasing *r*_step_ step input magnitudes reveal a threshold value for an output to be elicited in the case of the bistable system (solid curve). The response curve for the excitable system is shown in the dotted curve. The threshold D. Time responses (blue line) of three cases a, b and c with Δ*t* as the duration of the input pulse (red curve). E. Computing the response (final value) with increasing Δ*t* pulses of the unit magnitude reveal a minimum pulse duration for an output to be elicited in the case of the bistable system (solid curve). The response curve for the excitable system is shown in the dotted curve. F. Plot of the minimum pulse duration for different input pulse strengths.

The model we use is inspired by the classical FitzHugh-Nagumo set of equations [13, 14]. It consists of activator (*u*) and inhibitor (*v*) species coupled using positive and negative feedback loops (Fig 1A). The system dynamics obey the following two differential equations

$$\dot{u}=f\left(u,v\right)=-a_{1}u-a_{2}u\left(v-r\right)+\frac{a_{3}u^{2}}{a_{4}+u^{2}}+a_{5}$$ (1a)

$$\frac{1}{\varepsilon }\dot{v}=g\left(u,v\right)=-v+q_{1}u$$ (1b)

where the coefficients are all positive constants and *r* is the input signal (Section A.1 in S1 Appendix). A critical requirement for excitability is that *ε* ≪ 1 so that the effect of the inhibitor is significantly delayed as compared to the activator. When coupled with diffusion in space, these equations are sufficient to recreate the signalling patches and wave propagation observed in migrating cells [6, 10, 15–17].

To gain insight into the model and to introduce the idea of the threshold for activation, it is useful first to analyze the equations in phase-plane. Equating both *f*(*u*, *v*) and *g*(*u*, *v*) to zero, we obtain the two nullclines of the system. We assume that the parameters are such that the *u*-nullcline has a local minimum and maximum (Section A.2 in S1 Appendix) and that the *v*-nullcline, which is a straight line, intercepts the *u*-nullcline only once so that the system has a single equilibrium, which we label as (*u*_−_, *v*_0_), lying to the left of the local minimum (*u*_min_, *v*_min_) of the *u*-nullcline; see Fig 1B.

The input signal *r* consists of an external input and a stochastic variable (Section A.3 in S1 Appendix). A non-zero input signal *r* will displace the initial state as dictated by the magnitude of $\dot{u}$ and $\dot{v}$. However, the equilibrium is stable so that a small displacement of the state leads to a quick return to the equilibrium. If the initial displacement is sufficiently large, the positive feedback dominates the negative feedback and the state undergoes a large excursion beyond the local maximum of the *f* nullcline, from *u*_−_ to *u*_+_ as illustrated in Fig 1B, before returning to the equilibrium *u*_−_. This large excursion occurs because the initial set point is close to a Hopf bifurcation point, which exists at the minimum of the cubic nullcline. This can be seen by plotting the eigenvalues with lowering level of inhibition (*q*_1_) of the excitable system (S1 Fig). After a while, both eigenvalues change stability together, and are purely imaginary at the transition, indicating a Hopf bifurcation. Beyond this bifurcation point, the equilibrium is rendered unstable and the solitary large excursion becomes an oscillatory limit cycle.

As excitable systems require that *ε* ≪ 1, trajectories from *u*_−_ to *u*_+_ are nearly horizontal in the (*u*, *v*) plane. Suppose that this change occurs at near-constant level of *v* = *v*_0_. In the limit (*ε* = 0), this results in a one-dimensional differential equation. The above analysis of suprathreshold and subthreshold inputs is perhaps better visualized using this simplified system:

$$\dot{u}=-\left(a_{1}+a_{2}\left(v_{0}-r\right)\right)u+\frac{a_{3}u^{2}}{a_{4}+u^{2}}+a_{5}=f\left(u,v_{0}\right)$$ (2)

For a range of parameters (Section A.2 in S1 Appendix), this equation exhibits bistability, with three equilibria, two of which are stable and correspond to *u*_−_ and *u*_+_, while the unstable solution is *u*^⋆^ [18]. Thus, in the limit, the excitable system acts as a bistable system with the transition occuring between the two stable states of this first order system. Henceforth, we refer to this curve as the bistable nullcline (S1 Fig).

## Results

### A pulse input requires a minimum duration to elicit a response

The presence of two qualitatively different responses to inputs of varying sizes indicates the presence of a threshold for activation. Depending on the type of input applied, this threshold acts differently. In the context of cell migration, two types of inputs are pertinent. The first, a step input, indicates the introduction of a persistent chemoattractant concentration at the periphery of the cell. In this case the concentration is compared to the threshold and this is used to determine whether pseudopods should be formed. The second class of inputs consists of stochastic perturbations, which can be considered as high frequency noise. Conceptually, these signals can be approximated by a sequence of pulse inputs.

Whenever a step input is applied to an excitable system—that is, the level of the input changes from zero to some value *r*_step_ > 0 and remains there persistently—there exists a hard threshold value with respect to the magnitude of the stimulus. This implies that whether or not the system will respond is completely determined by the steady-state level of the stimulus. Phase-plane analysis of the bistable system gives us a clear picture of what the threshold value is for the step input case (Section A.4 in S1 Appendix). More particularly, for the bistable system, a response is triggered if and only if:

$$r_{\text{step}}>v_{0}-v_{\text{min}}$$

where *v*_0_ and *v*_*min*_ are the values indicated in Fig 1B, and *r*_step_ is the magnitude of the persistent stimulus. We simulated the associated system *f*(*u*, *v*_0_) with increasing magnitudes of *r*_step_. The maximal response exhibited a sharp transition after the input magnitude exceeded a given value; see Fig 1C. Simulations of the excitable system with 0 < *ε* ≪ 1 also displayed this threshold behavior, though the trajectories were not completely horizontal making the value of the threshold somewhat larger than *v*_0_ − *v*_min_ (Fig 1C).

When the external input is transient, as in a pulse, this threshold value no longer holds. That is, the magnitude of the stimulus is no longer sufficient to determine the response. From the phase-plane analysis it is clear that even if the magnitude of the pulse exceeds the step input threshold, the duration of the pulse must be sufficiently large for the state to cross the unstable steady state of the system (Section A.4 in S1 Appendix). This implies that there exists a minimum pulse duration (*t*_min_) below which no response is obtained, irrespective of the pulse magnitude, thus showing that the input level is not sufficient to characterize the threshold of the system. Though we cannot present a description of the threshold in terms of *r*, we can define a function *s*(*r*, Δ*t*), that reflects *the distance traversed by the state u during the duration of the pulse*. For a pulse input of magnitude *r*_pulse_ and duration Δ*t*, from Eq 2 we get:

$$s\left(r,\Delta t\right)=\int_{0}^{\Delta t}(-a_{1}u-a_{2}\left(v_{0}-r_{\text{pulse}}\right)u+\frac{a_{3}u^{2}}{a_{4}+u^{2}}+a_{5})dt$$

In this case, the bistable system with *f*(*u*, *v*_0_) responds if and only if:

$$s\left(r,\Delta t\right)>u^{⋆}-u_{-}$$

where *u*^⋆^ denotes the unstable equilibrium of the bistable system (*u*_−_ < *u*^⋆^ < *u*_+_). As *u*_−_ is the initial state, this implies that the bistable system responds if and only if, at the end of the pulse duration, the state *u* has crossed the unstable equilibrium of the system, irrespective of the magnitude of the input pulse. To illustrate this property we stimulated the system with signals with varying Δ*t* pulses but with the same magnitude. As shown in Fig 1D, the response eventually disappeared for sufficiently small Δ*t*. By calculating the response to pulses with gradually increasing Δ*t* we obtained the minimum pulse duration threshold beyond which a response can be triggered (Fig 1E).

Analytically, we can calculate the minimum pulse duration, *t*_min_, needed for a particular input magnitude by determining the time needed for the state to travel from (*u*_−_, *v*_0_) to (*u*^⋆^, *v*_0_) under an input pulse amplitude of *r*_pulse_. Integrating the bistable system *f*(*u*, *v*_0_) of Eq 2 we get:

$$t_{\text{min}}=\int_{u_{-}}^{u^{⋆}}(-a_{1}u-a_{2}\left(v_{0}-r_{\text{pulse}}\right)u+\frac{a_{3}u^{2}}{a_{4}+u^{2}}+a_{5}{)}^{-1}du$$

The value of *t*_min_ calculated by evaluating this integral numerically using the system parameters, matched the *t*_min_ obtained from simulations (Fig 1E). Note that *t*_min_ depends on the magnitude of the pulse, i.e. as expected, a greater input magnitude makes the minimum pulse duration smaller (Fig 1F).

### The threshold to pulse inputs can be decoupled from the hard threshold seen by step inputs

The minimum pulse duration criteria is instrumental in revealing the soft threshold, as the point-of-no-return for the state—*u*^⋆^ constitutes a part of the threshold manifold (the separatrix [11]) of the excitable system. To manipulate this threshold, we considered changes in the system that alter this minimum pulse duration. The critical time *t*_min_ is determined by two things: the distance between the initial set point and the unstable equilibrium (*u*^⋆^ − *u*_−_) and the rate of change of the state ($\dot{u}$). One obvious way of changing *t*_min_ is by altering the dynamics of the system by modifying the time scale. For example, doubling *f* in Eq 2 leaves the nullcline intact but halves *t*_min_. We focused, instead, on changes that affect the threshold by modifying the nullcline, without significant changes to the system dynamics. Specifically, we considered changes in the system that alter the *distance* between *u*_−_ and *u*^⋆^ to study how these affect the minimum pulse duration.

**Fig 2:**
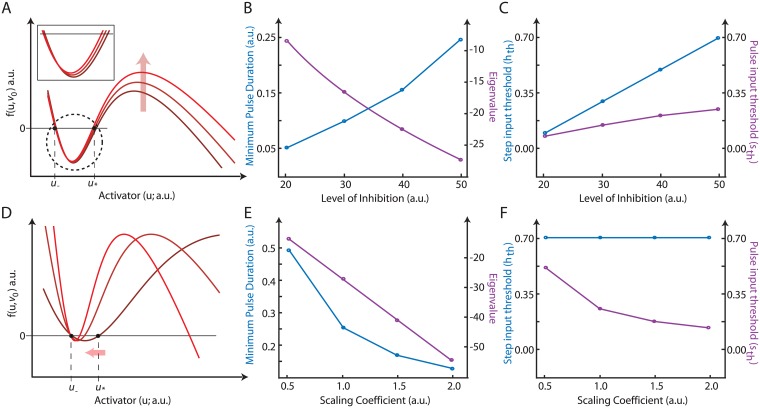
Modulating the minimum pulse duration. A. The bistable nullcline (*f*(*u*, *v*_0_)) plotted as a function of the state *u* (green curve), with increasing initial inhibition (*v*_0_) levels as indicated by the arrow. A zoomed in version of the critical region is shown in the box in order to highlight the raising of the nullclines. B. (blue) Graph plotting the minimum pulse duration needed to elicit a full response with increasing levels of inhibition. (purple) Location of eigenvalue corresponding to the new equilibrium state with each level of inhibition. Only one pertinent eigenvalue is shown. C. Graph plotting how the step (blue) and pulse (purple) input thresholds changed with altering level of inhibition. D. The bistable nullcline (*f*(*ku*, *kv*_0_)) plotted against *u* with increasing values of *k* causing a contraction of the nullcline as indicated by the red arrow (dark red: *k* = 0.5, lighter red: *k* = 1, light red: *k* = 1.5). E. (blue) Plot showing the minimum pulse duration needed to elicit a full response with increasing scaling coefficient *k*. (purple) Location of the eigenvalue corresponding to the new equilibrium state with each *k*. F. Graph plotting how the step (blue) and pulse (purple) input thresholds changed with altering scaling coefficient.

A change in *v*_0_ alters the distance between *u*_−_ and *u*^⋆^. More specifically, raising *v*_0_ is equivalent to lowering the bistable nullcline (*f*(*u*, *v*_0_)), increasing the critical distance that determines the pulse threshold (Fig 2A). We simulated the system with higher *v*_0_ levels by increasing the strength of the negative feedback *q*_1_ in Eq 1. These simulations showed that the minimum pulse duration needed to elicit a response is greater because of the higher threshold (Fig 2B). The stronger negative feedback gains also moved the eigenvalues of the excitable two-dimensional system farther away from the imaginary axis, representing greater stability margin (Fig 2B). In these simulations, increasing *v*_0_ led to higher step and pulse input thresholds, i.e. both *v*_0_ − *v*_min_ and *u*^⋆^ − *u*_−_ increased (Fig 2C).

Though the previous simulations suggest a link between stability margins and thresholds, we found situations in which an increase in the stability margin can accompany a smaller threshold. For example, scaling the variables from *f*(*u*, *v*_0_) to *f*(*ku*, *kv*_0_) with *k* > 1, compresses the nullcline (Fig 2D). Simulations of this system showed that the minimum pulse duration needed to trigger a response was smaller as the distance between *u*_−_ and *u*^⋆^ decreased without an appreciable change in dynamics indicating a smaller threshold (Fig 2E). However, the eigenvalues of the corresponding linearized excitable system moved farther away from the imaginary axis, denoting greater stability margin (Fig 2E). Most importantly, in this situation, increasing the variable scaling and compressing the nullclines, lowered the pulse input threshold *u*^⋆^ − *u*_−_, but *did not affect* the step input threshold *v*_0_ − *v*_min_ (Fig 2F) of the bistable system *f*(*u*, *v*_0_).

It is interesting to note from Fig 2F that the distance between *u*_−_ and *u*^⋆^ can be altered while keeping the *v*_0_ − *v*_min_ constant. This implies that the thresholds to the pulse and step inputs can be decoupled. Note that this does not suggest that there are two different thresholds for the system, but it does imply that the threshold manifests differently depending on the type of input. This can be thought of as a second threshold parameter for controlling the response. For this purpose, we introduce the terms *h*_th_ and *s*_th_ to denote the two forms of the threshold. More particularly, for the step input and the bistable system we must have:

$$r_{\text{step}}>h_{\text{th}}=v_{0}-v_{\text{min}}$$

and for the pulse input and the bistable system we must have:

$$s\left(r,\Delta t\right)>s_{\text{th}}=u^{⋆}-u_{-}$$

where *r*_step_ and *s*(*r*, Δ*t*) were defined above.

### The pulse threshold endows excitable systems with noise filtering capabilities

The fact that the threshold of excitable systems sets a limit on the duration of the input pulse needed to obtain a response suggests that noisy signals, which can be thought of consisting of short duration pulses, may not elicit a response from the system. To investigate the filtering properties of our system in the context of cell migration, we compared the response of the excitable system to that of a zero-order ultra-sensitive switch, an alternative signal amplification mechanism suggested for cell migration [19, 20]. To ensure a fair comparison, we tuned the output dynamics of the switch until the response time was comparable to that of the excitable system (Section A.5 in S1 Appendix). Identical stimuli, in which the input noise was modeled as a Gaussian, white noise process with zero mean and constant variance, were applied to both systems. In this comparison, the threshold of each system was chosen so that a step input of the same magnitude could just trigger both systems.

**Fig 3:**
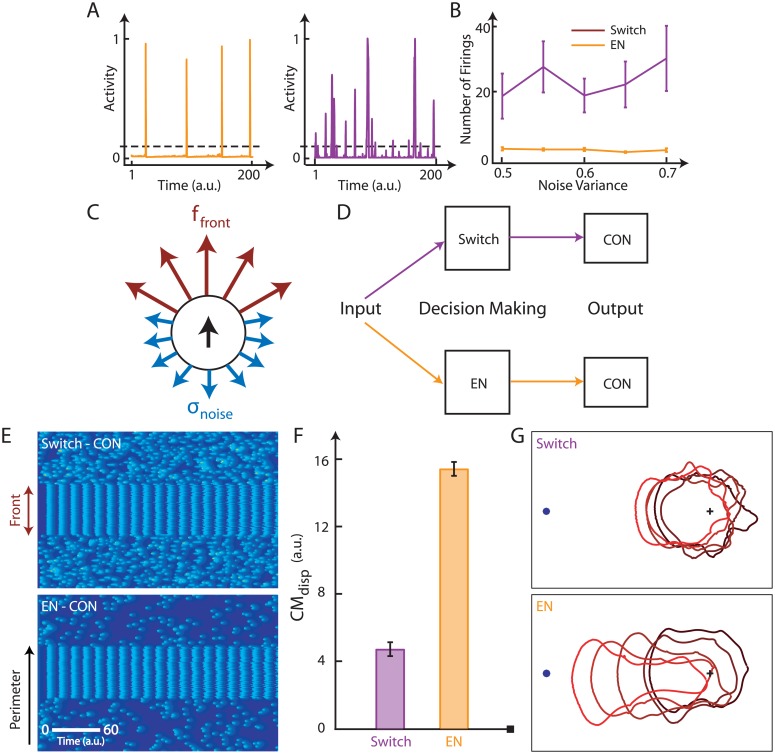
Benefits of an excitable system in directed migration. A. Examples of the responses of the excitable system (left) and switch (right). The responses have been normalized. B. The number of suprathreshold firings (peak ≥ 0.1, as indicated by the dashed black line in the example responses as a function of the noise variance for both systems that occurred in 200 simulation time units. Error-bars were calculated using 10 simulations. C. Schematic of simulations of cell migration, in which a uniform force (*f*_*front*_) is applied to the front of the cell while the back fires stochastically, with *σ*_*noise*_ serving as the input for both systems. D. The input-output pathway where the decision making occurs through either a switch (purple) or an excitable system (EN, orange). The output of both is fed to a common cytoskeletal excitable network (CON) from which the output activity is considered. E. One dimensional simulations shown via kymographs where activity is indicated through patches that are graded in color from blue to yellow corresponding to low to high activity respectively. The front of the cell (indicated in brown) oscillates identically for both cases (top—switch, bottom—excitable network) while the rest of the perimeter fires stochastically depending on the output from D. F. The kymographs in E were used to obtain the net displacement of the center of mass of the cell for both cases. Error-bars were calculated using 10 simulations. G. Level set simulations for the kymographs indicated in E, shown through overlayed frames. The blue dot indicates the chemoattractant source while the position of the cell boundary with time is shown through lighter shades of red. The initial center of the cell is denoted by the black plus sign which acts as a fixed spatial fiduciary.

For a particular level of threshold, simulations were carried out with different noise variance levels and the outputs of the systems were compared by counting the number of firings (Section A.3 in S1 Appendix). For all variances considered, the excitable network showed fewer firings than the ultrasensitive switch (Fig 3A). Moreover, the number of firings fluctuated with noise variance for the switch, but remained relatively constant for the excitable system (Fig 3B). Though there existed a maximum number of firings that could occur in a specific amount of time for the excitable system because of the refractory period, no such limitation existed on the number of firings of the switch. However, as we show in the next section, even with the incorporation of a refractory period for the ultra-sensitive switch, the excitable system served as a more robust noise filter.

### The excitable system threshold enables faster chemotaxis

To compare the efficacy of an excitable network to other switching mechanisms as a means for providing a threshold for activation in directed cell migration, we carried out simulations to compare the responses of a switch and an excitable system on a discretized one-dimensional cell boundary. In these simulations, the equations of Eq 1 were modified to add diffusion terms (Section A.6 in S1 Appendix).

The simulations were set up as shown in the schematic of Fig 3C, where a uniform force was applied at the front of the cell and the back was allowed to fire stochastically through one of the candidate systems. To ensure fair comparison metrics, the stimulus was first processed through one of the candidate circuits before being fed into a second excitable system (Fig 3D). The latter models the action of the actin cytoskeleton, which has been shown to behave as an oscillatory excitable system [6, 21] (Section A.7 in S1 Appendix). This implies that the decision to fire or not is taken by the candidate system while the actual firing comes from a common system. We used this method to guarantee that the movement response is the same for both systems once the threshold is crossed. It also ensures the existence of a similar refractory period for the actin response for both systems.

To compare the effectiveness in directed migration for both the systems we put the cytoskeletal excitable network into the oscillatory limit cycle mode at the front of the cell. This was done as the limit cycle enabled the fastest turnaround of activity in the front of the cell, i.e. the maximum possible pull towards the front, while also ensuring the same front force for both the systems. In this setting, the noise filtering properties of the back of the cell were compared, as the system with greater filtering capabilities will result in faster migration.

As in seen in Fig 3E, one-third of the perimeter oscillated and formed the front of the cell as shown in the kymographs, while the rest of the perimeter fired in random patches for both systems. The kymograph activity was averaged with highest weight in the front region, while the back had a negative weight. Integrating this we obtained the net force that the cell experienced, towards the chemoattractant source. This force was passed through a viscoelastic model of the cell [22] to obtain the net displacement of the center of mass of the cell (CM_disp_).

We carried out a number simulations for each system using the same threshold and the same level of noise. As seen in the kymographs, the back of the cells regulated by the excitable network fired less often than those of cells using the hard switch. As a result, the cells that were controlled by the excitable network thus moved further up the gradient than those controlled by the switch (Fig 3F). Level set simulations were carried out [22] using the activity from the kymographs. As seen in Fig 3G, the cells governed by the excitable network moved closer to the chemoattractant source in the same amount of time. The level set simulations are provided in S1 Video.

### The decoupled thresholds of the excitable system allow finer control in chemotaxis

**Fig 4:**
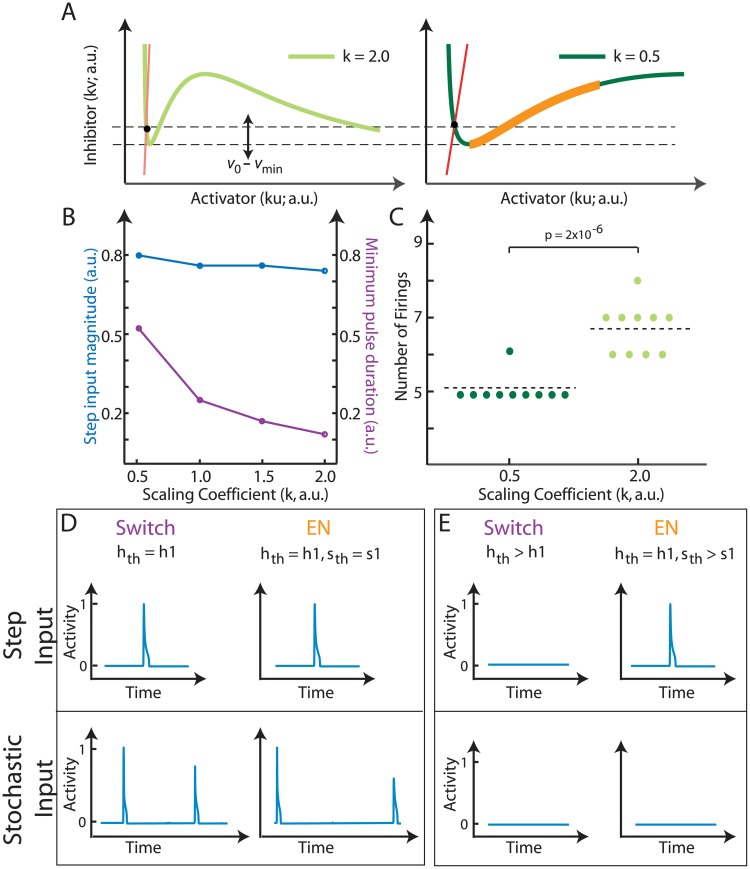
The control mechanism using the decoupled thresholds. A. The excitable system (*f*(*ku*, *kv*)) represented in phase space. The activator nullcline plotted for *k* = 0.5 (right) and *k* = 2 (left). The inhibitor nullcline is denoted in red. The constant step input threshold is indicated between the two dashed lines. The separatrix for one system is shown in orange for reference. B. Quantification of the magnitude of the step needed to elicit a response (blue curve) and the minimum pulse duration needed to elicit a response (purple curve) for the different scaling factors in an excitable system. C. Bee-swarm plot of the number of suprathreshold firings for the cases in A shows a significant change in the firing rate (p-value obtained from student’s t-test). Number of firings were calculated in 200 simulation time units for a noise variance of 0.5. D. Response to a step input. (left) Output from the switch (purple, from Fig 3D, threshold value indicated as *h*_th_) to a step input (top) and a stochastic input (bottom). (right) Output from the excitable system (orange, from Fig 3D, step and pulse threshold values indicated as *h*_th_ and *s*_th_ respectively) to a step input (top) and a stochastic input (bottom). E. Response to a stochastic input. Same output scheme as in D just with an increased step input threshold for the switch and an increased pulse input threshold for the excitable system.

Apart from the filtering properties of the pulse threshold, we have shown that bistable systems allow a decoupling of the thresholds to step and pulse inputs. The same decoupling extends to excitable systems where one can keep the step threshold (*h*_th_) constant, while altering the distance between *u*_−_ and *u*^⋆^ in the excitable system to improve upon its filtering capabilities, i.e. tuning out noise effects while maintaining the same hard threshold level for the system. We explored this possibility as a means of improving chemotactic efficiency. We scaled the activator and inhibitor equations of the system (from *f*(*u*, *v*), *g*(*u*, *v*) to *f*(*ku*, *kv*), *g*(*ku*, *kv*)) thereby conserving the difference between the set point and the minimum (*v*_0_−*v*_*min*_) (Fig 4A) but altering the distance between *u*_−_ and *u*^⋆^. Simulations were done to reveal the step and pulse thresholds and as expected, the change in the *h*_th_ threshold was negligible as compared to the change in the minimum pulse duration (Fig 4B). As a result, the number of firings of the system decreased significantly when the critical distance (*s*_th_) increased (Fig 4C).

To illustrate the advantage of having a decoupled pulse threshold in addition to a hard threshold, we considered both persistent and stochastic perturbations using the scheme from Fig 3. Simulations of the systems showed that persistent suprathreshold stimuli gave rise to nearly identical responses (Fig 4D, top). Similarly, stochastic stimuli gave rise to a series of firings in both systems (Fig 4D, bottom). We next considered the possibility of eliminating the presence of noise-driven firings by raising the system thresholds.

As desired, this removed the stochastic-driven firings in both systems, but also eliminated the response due to a step in the ultrasensitive switch (Fig 4E). In contrast, the presence of both *h*_th_ and *s*_th_ in the excitable system provided a means for discriminating between the two types of stimuli. We raised *s*_th_ while leaving *h*_th_ intact, and simulations showed that this selectively attenuated the response due to stochastic fluctuations, while maintaining that due to the step stimulus (Fig 4E).

**Fig 5:**
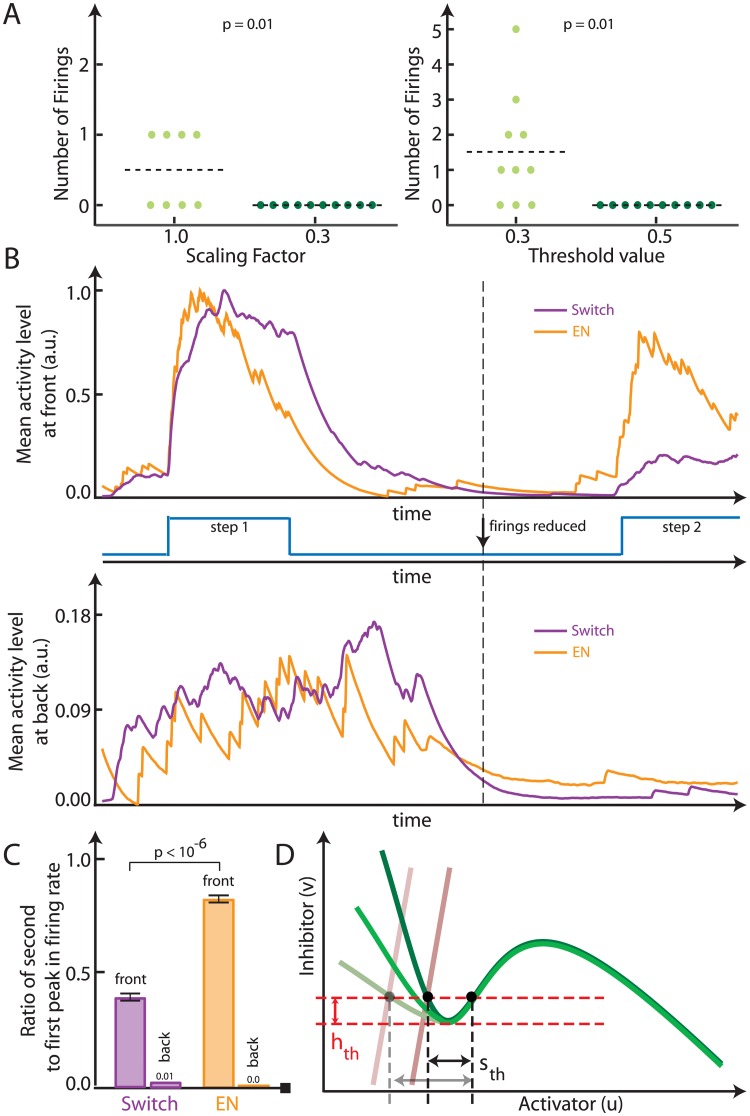
Control of chemotaxis signaling. A. Lowering the number of firings of the excitable system (left) using the scaling factor *k*, and of the ultra-sensitive switch (right) using the hard threshold value. The scaling factor and the threshold value were increased till zero firings were obtained for a noise variance of 0.2. Number of firings were calculated in 200 simulation time units. B. Illustration of the effect of the control mechanism on cell migration response. Quantification of the mean activity at the front (top) and back (bottom) of a migrating cell using the control mechanism (middle). The step inputs (middle) are aligned in time with the front and back response plots. The back activity is presented as a percentage of the total front response, which is normalized. The step response magnitude chosen for this illustration was 0.3. After a point in time (vertical dashed line) the firings were reduced by altering the scaling factor for the excitable system and the threshold value for the switch as dictated by A. C. The number of firings during the two step responses—before and after firings were reduced, were quantified. Errorbars were from 10 simulations. The ratio between the two firing rates are presented for both the front and back of the cell. The step magnitude chosen here was 0.4 for a *q*_1_ = 30. D. The excitable system nullclines (green—activator, brown—inhibitor) plotted in phase space, showing the ideal scenario where just the pulse input threshold (dashed black lines) is altered while the step input threshold (dashed red lines) and the rest of the nullcline remains conserved.

The ability to attenuate noise signals selectively suggests the possibility of designing a conceptual system to achieve finer control and greater efficiency during chemotaxis. In particular, we suggest a three-step mechanism. First, we would apply a chemoattractant gradient to the cell and observe the number of firings at the side furthest from the chemoattractant source. Second, we would remove the chemoattractant stimulus and alter the threshold to reduce the number of stochastically-induced firings. The method of reducing threshold would differ for the switch and the excitable system (Fig 5A). Finally, we would reintroduce the stimulus to achieve the same response but now with lower activity at the rear.

To test the efficacy of this scheme, we developed a simulation for spatially graded systems as described above, under the assumption that the gradient is interpreted by either an excitable system or an ultrasensitive switch. In both cases the chemoattractant concentration is used to lower the threshold at the front and the response of the network leads to increased cytoskeletal activity [10, 23].

Our simulations showed that, after application of the gradient, there was a steep rise in activity in the region of highest chemoattractant concentration (Fig 5B, step 1). At the rear, some stochastically-driven firings took place, but overall there was lower activity than at the front. In the second part of the simulation, the switch and excitable network thresholds (*s*_th_) were adjusted so that both systems showed fewer firings (Fig 5B, firings reduced). The levels were adjusted as dictated by Fig 5A, so as to match the reduced number of firings. After the chemoattractant gradient was reintroduced into the cell (Fig 5B, step 2), the level of rear activity was reduced significantly for both systems (Fig 5B, bottom); however, at the front, the excitable system had higher activity level than the ultra-sensitive switch (Fig 5B, top). The difference between the two systems is due to the fact that increasing the threshold value for the switch lowered the response to both stochastic and persistent stimuli. Specifically, the switch lost its step response to a range of input magnitudes. For the excitable system, however, increasing *s*_th_ had little effect on *h*_th_, thus minimizing the effect on the step response. As we scaled the nullclines to alter *s*_th_ in the excitable system case, the maximum response (*u*_+_) was altered. Thus, for quantification, instead of mean activity, we calculated the number of firings at the front and back—before and after thresholds were altered for both systems. The ratio of the second and first peaks are shown in Fig 5C. While both systems show almost no firings at the back of the cell, at the front the response of the excitable system to the second step is significantly larger.

## Discussion

While the presence of a threshold for activation in this cascade has been well documented in studies of chemotaxis of amoeboid cells [5, 20, 24, 25], it is only recently that it has been appreciated that this threshold arises from the existence of an excitable network [9, 10, 26–32]. Recent advances in cell biology have allowed acute genetic perturbations of the signaling network that lower this threshold, leading to greater signaling activity, increased actin polymerization along the cell cortex, and faster moving cells [17, 33].

Models of how cells interpret external chemoattractant concentrations to direct cell migration suggest that cells process the information through a cascade of interconnected networks [9, 32, 34–37]. The chemoattractant signal is first processed by a Local-Excitation, Global-Inhibition (LEGI) scheme [19]. Gradients of chemoattractant lead to a persistent signal at the front and rear of the cell that alter the threshold of a signaling transduction excitable network (STEN). Persistent signals from LEGI lead to lowered or raised thresholds at the front or back, respectively, lead to spatially biased activity [10]. This network then controls a cytoskeletal oscillatory network (CON) that is at the borderline between excitable and oscillatory [6, 21].

The existence of an excitable system at the center of the signaling network controlling movement explains a number of features of the chemotactic response, including the presence of a threshold for activation and high amplification of the external stimulus, as well as why cells move in the absence of gradients, but using seemingly identical biochemical machinery. Importantly, the system must respond to a combination of persistent and stochastic signals. The former, in the context of chemoattractant gradients; the latter for random migration. Our results explain why an excitable system is ideal. Because of the presence of a threshold, the front fires frequently while the back remains relatively quiescent causing the cell to move in the desired direction. Importantly, the back should remain robustly impervious to stochastic fluctuations for increased efficiency. As shown here, excitable systems are less affected by high levels of noise than other systems proposed in literature to account for the signal amplification such as the zero-order ultrasensitive switch [20, 38, 39].

The notion that excitable systems have some noise-filtering benefits is not completely new. Previously, Samoilov and coworkers studied stochastic resonance in excitable networks, in which sinusoidal inputs of varying frequency are applied to the system and result in repeated oscillatory excursions [40]. Stochastic resonance is observed in excitable systems that are operating at or near a Hopf bifurcation point. While there is some evidence that the cytoskeletal network (CON) of *Dictyostelium* cells is operating at or near an oscillatory point [6, 21], it appears that relatively few cells have a signaling network that operates in an oscillatory mode [17].

The presence of a threshold manifold in excitable systems has been previously proposed [11]. Here we demonstrated that that this manifold provides the system with a different representation of threshold for persistent and pulse stimuli. The pulse input threshold has a minimum time duration criteria, allowing it to ignore high-frequency noise-like signals. Moreover, for the excitable system, the step input threshold and the pulse input threshold can be decoupled from one another. These decoupled thresholds present an advantage by which one can selectively tune the response of the cell to noise inputs while conserving its response to chemoattractants. For systems without a decoupled threshold, the noise inputs can be attenuated but the response to a range of step inputs will simultaneously be lost. We constrained ourselves to a FitzHugh-Nagumo type system, where we had to scale the nullclines to alter the pulse threshold—which in turn changed the maximal response. In higher order systems it is possible to preserve the rest of the nullcline and just alter the critical distance as illustrated in Fig 5D.

With advances in optogenetic approaches for biological perturbations [41], it may be possible to explore the pulse threshold of the cell to investigate the genetic circuitry regulating this threshold. If the decoupled thresholds controllers can be revealed then one can potentially engineer cells that can undergo highly controlled chemotaxis.

## Supporting information

S1 Appendix Mathematical modeling details. (PDF)

S1 FigExcitable system model analysis.A. The excitable system in phase space with the activator and inhibitor nullclines in green and red respectively. The black arrow shows how the inhibitor nullcline changes when the level of inhibition (*q*_1_) is decreased. B. The eigenvalues plotted for lowering level of inhibition from A. Real part of the eigenvalues change stability together, when both eigenvalues are purely imaginary (vertical dashed line), indicating the Hopf bifurcation point. C. The bistable nullcline *f*(*u*, *v*_0_) plotted as a function of the state *u* (green curve). The trajectories of the activator state are shown at different concentrations. A full response is obtained when the initial state (*u*_−_) can follow the green arrows ($\dot{u}>0$) to reach *u*_+_ without encountering the red arrow where $\dot{u}<0$. The step input *r*_*step*_ causes the nullcline to be raised vertically as shown (shaded grey arrow) and the threshold value is indicated (*h*_*th*_). Note, the nullcline does not move perfectly vertically as shown. A vertical projection is considered for easier understanding. D. The bistable nullcline (*f*(*u*, *v*_0_)), plotted against the state *u*, is raised vertically when *r*_*pulse*_ is applied. The dashed green curve along with the dashed trajectories depict the situation during the pulse input while the solid green curve and the solid trajectories denote the situation before and after the pulse. As the pulse is applied, the state *u* starts following the dashed green trajectories towards *u*_+_. Case a. If the pulse duration is not sufficient for the dashed brown trajectory to cross *u*^⋆^, the state is forced to return back to *u*_−_ (solid brown arrow). Case b. If the pulse duration is sufficient for *u* to cross *u*^⋆^ (dashed brown arrow) then even after the pulse is withdrawn (solid brown arrow) the state can reach *u*_+_. Case c. If the pulse duration causes the state to cross *u*_+_ (dashed brown arrow), once the pulse is removed, it returns to settle at *u*_+_ (solid brown arrow). The critical distance is indicated as *s*_th_.(EPS)

S1 Video Simulations of directed cell migration. Level set simulations of the cell boundary as outlined in Fig 3. The cells start from the red line and move towards the black line. The cell on the top is controlled by the excitable network and the one on the bottom is controlled by a switch as indicated. The force generated with protrusions is color graded from red to blue. The space dimension is provided.
(MOV)
