# Supplementary material for: The threshold of an excitable system serves as a control mechanism for noise filtering during chemotaxis
Source: PLoS One. 2018 Jul 30;13(7):e0201283. doi: 10.1371/journal.pone.0201283 (PMC6066244; doi:10.1371/journal.pone.0201283)
Supplement: S1 Appendix — (PDF) [file pone.0201283.s001.pdf]

A Appendix

A.1 Model equations

The classical FitzHugh-Nagumo equations are of the form:

$$\frac{\mathrm{d}u}{\mathrm{d}t} = -u(u-a)(u-1) - v \tag{3a}$$

$$\frac{\mathrm{d}v}{\mathrm{d}t} = \varepsilon(-v + bu) \tag{3b}$$

where u and v represent the activator and inhibitor, respectively, while $0 < a < 1$ and b are constants.

The inhibitor equation is used without any change in the model system of 1. After simplification, the activator equation is as follows:

$$\frac{\mathrm{d}u}{\mathrm{d}t} = -u^3 + u^2(1+a) - au - v.$$

The state variables in the FitzHugh-Nagumo equation represent electrical signals, while those used in the biological model are protein/lipid concentrations. For this reason, this equation is altered to make the system realistic biologically with the following assumptions:

1. The cubic and linear terms represent self degradation and can be grouped into one term dependent on u.
2. The '$-v$' term, representing the negative feedback from the inhibitor, is made dependent on u so as to ensure that the concentrations remain greater than zero.
3. The quadratic term, represents positive feedback, but can grow unboundedly and thus a saturation effect is added to the term.
4. A basal level of activation is added to the equation to ensure non-zero concentrations.

Under these approximations the equation takes the form:

$$\frac{\mathrm{d}u}{\mathrm{d}t} = -m_1(1 + m_2 v)u + m_5 + \frac{m_3 u^2}{m_4 + u^2}.$$

With the addition of an external input signal r to the above equation, the differential equation describing the concentration of the activator can be written as:

$$\frac{\mathrm{d}u}{\mathrm{d}t} = -a_1(1 + \bar{a}_2 v)u + a_5(1 + \bar{a}_6 \bar{r} u) + \frac{a_3 u^2}{a_4 + u^2}.$$

The first two terms describe the decrease in the concentration, which consists of a basal rate (a_1) plus an inhibitor-dependent rate ($a_1 \bar{a}_2 v$) which represents the effect of negative feedback. The next two terms represent increases in the concentration — again, because of a basal rate (a_5) which increases in a positive-feedback manner in the presence of an external input, $\bar{r}$: ($\bar{a}_6 \bar{r} u$). The final term represents nonlinear positive feedback with Hill coefficient two. Note that this positive feedback may represent the effect of a double-negative feedback loop [17]. As shown below, it will be convenient to rewrite this as follows

$$\begin{aligned}\frac{\mathrm{d}u}{\mathrm{d}t} &= -a_1 u - (a_1 \bar{a}_2 v + \bar{a}_5 \bar{a}_6 r)\, u + \frac{a_3 u^2}{a_4 + u^2} + a_5 \\ &= -a_1 u - a_1 \bar{a}_2 \left(v - \bar{a}_5 \bar{a}_6 \bar{r}/(a_1 \bar{a}_2)\right) u + \frac{a_3 u^2}{a_4 + u^2} + a_5.\end{aligned}$$

We define $a_2 = a_1 \bar{a}_2$ and $r = \bar{a}_5 \bar{a}_6 \bar{r}/(a_1 \bar{a}_2)$, leading to the equations describing the complete system given in Eq.1.

A.2 Nullclines, equilibria and stability

To solve for the u-nullcline, we set $\frac{du}{dt} = 0$ and obtain:

$$\begin{aligned} v - r &= \frac{1}{a_2 u}\left(-a_1 u + \frac{a_3 u^2}{a_4 + u^2} + a_5\right) \\ &= -\frac{a_1}{a_2}\frac{u^3 - \gamma_1 u^2 + \gamma_2 u - \gamma_3}{u(\gamma_2 + u^2)} \end{aligned}$$

where

$$\gamma_1 = \frac{a_3 + a_5}{a_1}, \quad \gamma_2 = a_4, \quad \text{and} \quad \gamma_3 = \frac{a_4 a_5}{a_1}$$

are all positive constants. We want this nullcline to have the characteristic "upside-down-N" shape. To place conditions on the coefficients that accomplish this, we differentiate and set to zero:

$$\frac{\mathrm{d}}{\mathrm{d}u}\left(\frac{u^3 - \gamma_1 u^2 + \gamma_2 u - \gamma_3}{u(\gamma_2 + u^2)}\right) = \frac{\gamma_1 u^4 + (3\gamma_3 - \gamma_1\gamma_2)u^2 + \gamma_2\gamma_3}{u^2(\gamma_2 + u^2)^2}.$$

Clearly the denominator is positive for $u > 0$. The numerator has real solutions for u^2 if and only if

$$0 < \Delta = (3\gamma_3 - \gamma_1\gamma_2)^2 - 4\gamma_1\gamma_2\gamma_3 = (9\gamma_3 - \gamma_1\gamma_2)(\gamma_3 - \gamma_1\gamma_2)$$

which is satisfied if $\gamma_3 < \gamma_1\gamma_2/9$, or $\gamma_3 > \gamma_1\gamma_2$. In the original variables, this amounts to

$$\frac{a_4 a_5}{a_1} < \frac{a_4}{9}\frac{a_3 + a_5}{a_1}, \quad \text{or} \quad \frac{a_4 a_5}{a_1} > a_4\frac{a_3 + a_5}{a_1}.$$

The latter is impossible as all variables are positive. Thus, we need: $8a_5 < a_3$, which is indicative of a sufficiently strong positive feedback term. In this case the minimum for the nullcline occurs at

$$\begin{aligned} u_{\min}^2 &= \frac{\gamma_1\gamma_2 - 3\gamma_3 - \sqrt{(9\gamma_3 - \gamma_1\gamma_2)(\gamma_3 - \gamma_1\gamma_2)}}{2\gamma_1\gamma_2\gamma_3} \\ &= a_1\frac{a_3 - 2a_5 - \sqrt{a_3(a_3 - 8a_5)}}{2a_4 a_5(a_3 + a_5)}. \end{aligned}$$

Denote by $v_{\min}$ the corresponding value of v for this $u_{\min}$. Note that though there is no guarantee that $v_{\min} > 0$, this can be ensured by suitable choice of $r > 0$. In the obvious manner, we define

$$u_{\max}^2 = a_1\frac{a_3 - 2a_5 + \sqrt{a_3(a_3 - 8a_5)}}{2a_4 a_5(a_3 + a_5)}$$

with $v_{\max}$ denoting the corresponding local maximum value of v.

The v-nullcline, of course, is a straight line of slope q_1 and passing through the origin. The possible equilibria are the solutions of

$$q_1 u - r = -\gamma_0\frac{u^3 - \gamma_1 u^2 + \gamma_2 u - \gamma_3}{u(\gamma_2 + u^2)}$$

where $\gamma_0 = a_1/a_2$. This leads to a quartic equation for possible equilibria:

$$q_1 u^4 + (\gamma_0 - r)u^3 + (q_1\gamma_2 - \gamma_0\gamma_1)u^2 + \gamma_2(\gamma_0 - r)u - \gamma_0\gamma_3 = 0.$$

Note that the u^3 and u coefficients share the same sign, whereas the u^4 and constant terms are positive and negative, respectively. This means that, if the u^2 and u^3 coefficients have the same

sign, then there is exactly one real, positive solution by Descartes's rule of signs. This condition can be ensured if q_1 is sufficiently high:

$$q_1 > \frac{\gamma_0\gamma_1}{\gamma_2} = \frac{a_3+a_5}{a_2a_4}$$

and r is not too large: $r < \gamma_0$. We denote this equilibrium (u_-, v_0). Of course, for small q_1:

$$q_1 < \frac{a_3+a_5}{a_2a_4} \quad \text{and} \quad r > \gamma_0,$$

the resultant solution has the v-nullcline intersecting the u-nullcline in the right branch, and this results in an equilibrium to the right of $u_{\max}$.

Lastly, we investigate conditions for stability. The Jacobian of the system is given by

$$J = \begin{bmatrix} -a_1 - a_2(q_1u_- - r) + \frac{2a_3a_4u_-}{(a_4+u_-^2)^2} & -a_2u_- \\ \varepsilon q_1 & -\varepsilon \end{bmatrix}.$$

The (1,1)-element equals

$$-\frac{a_3u_-^2(u_-^2-a_4)+a_5(a_4+u_-^2)^2}{u_-(a_4+u_-^2)^2}.$$

The numerator of this expression is

$$(a_3+a_5)u_-^4 + a_4(2a_5-a_3)u_-^2 + a_4^2a_5$$

which is positive if

$$\begin{aligned} u_-^2 &< \frac{a_4(a_3-2a_5)-\sqrt{(a_4(2a_5-a_3))^2-4(a_3+a_5)a_4^2a_5}}{2a_4^2a_5(a_3+a_5)} \\ &= \frac{(a_3-2a_5)-\sqrt{a_3(a_3-8a_5)}}{2a_4a_5(a_3+a_5)} \end{aligned}$$

in which case the trace is negative, the determinant is positive, and hence the equilibrium is stable.

A.3 Noise filter comparison metrics

To compare the noise filtering capabilities of the excitable system and the ultrasensitive switch, a stochastic variable n was added to the the input signal r from equations Eq.1a and Eq.4. This n was modelled as follows:

$$\frac{\mathrm{d}n}{\mathrm{d}t} = -n + \sigma N(0,1)$$

where $N(0,1)$ is a normal random variable with zero mean and unit variance. As this too is modeled as a biological concentration, sub-zero values were not allowed. σ was chosen as a constant between 0.2 and 0.5.

In this comparison, the threshold variable θ in Eq.4 was chosen such that $\theta = h_{\text{th}}$, where h_{th} is the hard threshold for the excitable system. The response was then normalized to the maximum response (s_t in Eq.4) for both systems. The number of firings were counted as the number of output spikes with a peak greater than 0.1.

A.4 Phase plane analysis contrasting step and pulse responses

A.4.1 The step input

We begin by considering the effect of a persistent stimulus (step input) on excitable system dynamics. As we are concerned with the initial state transition from u_- to u_+, we need only consider the effect on the bistable system $\dot{u} = f(u, v_0)$. Note that increasing r from 0 to r_{step} in Eq.2 raises the activator nullcline in phase space (S1 Fig C). The system cannot remain with $u = u_-$ as this is no longer an equilibrium of the altered system. Rather, as $f(u_-, v_0) > 0$, the level of u increases. Whether it can reach u_+ depends on the amount by which the $f(u, v_0)$ has been raised.

If the change is sufficiently small so that $\dot{u} = f(u_{\min}, v_0 + r_{\text{step}}) < 0$, the state will not move significantly. In this case, u stops its transition once it encounters the region where $\dot{u} < 0$ (red arrows in S1 Fig C) and settles at the new equilibrium, far from u_+. On the other hand, if the stimulus is sufficiently large such that $\dot{u} = f(u_{\min}, v_0 + r_{\text{step}}) > 0$, there is no $\dot{u} < 0$ region between u_- and u_+ and the state settles at the high level. The boundary between these two behaviors thus gives us the threshold for the system in the case of the step input.

A.4.2 The pulse input

We again consider the corresponding bistable system. Suppose that we apply a stimulus that raises the bistable nullcline sufficiently so that the system begins to transition from u_- to u_+. If the magnitude of r is greater than $v_0 - v_{min}$ then, in the step case, this represents a suprathreshold input.

As above, the addition of the stimulus creates a region between the old and new nullcline positions where $\dot{u} > 0$ (green dashed arrows) which causes the state to increase (S1 Fig D). If the stimulus is transient as in the case of a pulse (r_{pulse}), the magnitude of this stimulus is necessary but not sufficient to guarantee a transition. When the stimulus is removed at $t = \Delta t$, the nullcline shifts back into its initial position reestablishing regions in which the trajectory moves to the left ($\dot{u} < 0$; red solid arrows, S1 Fig D). If at time Δt the state u has not crossed $u^\star$, then the state is in one of these regions and returns towards the previous equilibrium (case a, S1 Fig D). Alternatively, if the state has moved beyond $u^\star$ then it continues to increase towards the higher equilibrium (case b, S1 Fig D). A third possibility exists in which at $t = \Delta t$, the state has increased beyond u_+. In this case, removal of the pulse leads to a decrease in u towards u_+. This trajectory shows as a transient overshoot (case c, S1 Fig D).

A.5 Model of the ultrasensitive switch

The equation definiing the ultra-sensitive switch was taken from Wang et al. [20]. This is given by:

$$\frac{\mathrm{d}s}{\mathrm{d}t} = \rho\left(\frac{r(s_t - s)}{k_m + (s_t - s)} - \frac{\theta s}{k_m + s}\right) \tag{4}$$

where s represents the output state of the switch, r represents the input signal, θ is the threshold for the system and ρ, k_m and s_t are constants.

The value of k_m was taken from Wang et al. The constants ρ and s_t were adjusted such that the output dynamics of the switch and the excitable system were similar. To ensure this, we created the following metrics:

- The parameter s_t denotes the maximum possible response magnitude of the system. This was made equal to the maximum value attained by the activator for the excitable system when a unit magnitude step input was applied.
- The parameter ρ determines the response time of the system. This was made such that an unit magnitude pulse of unit duration could attain the exact same output magnitude for both systems.

A.6 Reaction-diffusion equations

For simulation in one-dimension, the equation system of Eq.1 was replaced by partial differential equations that modeled the diffusion of the system as well. These equations are given as:

$$\frac{\partial u}{\partial t} = D_u \nabla^2 u + f(u,v) \tag{5a}$$

$$\frac{\partial v}{\partial t} = D_v \nabla^2 v + g(u,v) \tag{5b}$$

The cell boundary was discretized into 300 points with diffusion modeled using the central-difference approximation.

A.7 The cytoskeletal network

The cytoskeletal network operating downstream of the decsion-making system in Fig. 3A1 was modeled using the same set of equations as the excitable system:

$$\frac{\mathrm{d}u_c}{\mathrm{d}t} = -a_{1c}u_c - a_{2c}u_c(v_c - r_d) + \frac{a_{3c}{u_c}^2}{a_{4c} + {u_c}^2} + a_{5c} \tag{6a}$$

$$\frac{1}{\varepsilon_c}\frac{\mathrm{d}v_c}{\mathrm{d}t} = -v_c + q_{1c}u_c \tag{6b}$$

where u_c and v_c corresponds to the activator and inhibitor respectively of the cytoskeletal system. The input r_d was the response obtained from the decision making system. The values of the constants are indicated in A.8.

A.8 Table of simulation parameters

Excitable Network		Switch		CEN	
a_1	0.167	ρ	20	a_{1c}	0.167
a_2	16.67	s_t	5.5	a_{2c}	16.67
a_3	167	k_m	0.01	a_{3c}	167
a_4	1.44	θ	h_{th}	a_{4c}	1.44
a_5	1.47			a_{5c}	1.47
$\varepsilon(bistable)$	0			ε_c	0.1
$\varepsilon(excitable)$	0.03			q_{1c}	20
q_1	50				
D_u	0.2				
D_v	1				
